# Early and mature *Achromobacter xylosoxidans* biofilm in cystic fibrosis and non-cystic fibrosis isolates: dynamics and response to clinically relevant antibiotics

**DOI:** 10.1016/j.bioflm.2026.100375

**Published:** 2026-06-15

**Authors:** Vincent Jean-Pierre, Pauline Sorlin, Catherine Dunyach-Remy, Florian Salipante, Raphaël Chiron, Jean-Philippe Lavigne, Cassandra Pouget, Hélène Marchandin

**Affiliations:** a HydroSciences Montpellier, Université de Montpellier, CNRS, IRD, Service de Microbiologie et Hygiène Hospitalière, CHU de Nîmes, Montpellier, 34093, France; b Hospital Hygiene and Infection Control Team, Hospital Center Laval, Laval, 53000, France; c VBIC, Univ Montpellier, INSERM, Department of Microbiology and Hospital Hygiene, CHU Nîmes, Nîmes, France; d Service de Biostatistique, Épidémiologie, Santé Publique, et Information Médicale, Université de Montpellier, CHU Nîmes, Nîmes, 30900, France; e HydroSciences Montpellier, Université de Montpellier, CNRS, IRD, Centre de Ressources et de Compétences de la Mucoviscidose, CHU de Montpellier, Montpellier, 34093, France

**Keywords:** *Achromobacter xylosoxidans*, Biofilm, Early adhesion, Cystic fibrosis, Microfluidic flow system, Antibiofilm, Antibiotic

## Abstract

*Achromobacter xylosoxidans* is an opportunistic pathogen in both cystic fibrosis (CF) and non-CF patients, in whom biofilm formation contributes to bacterial persistence and antibiotic tolerance. This study aimed to characterize early and mature biofilm formation in 57 clinical *A. xylosoxidans* isolates using complementary and physiologically relevant approaches and to compare biofilm phenotypes according to isolate origin (CF/non-CF). Early adhesion was assessed using the Biofilm Ring Test®, mature biofilm viable biomass was quantified under static conditions by colony-forming units counts, and biofilm dynamics were analyzed in a continuous-flow microfluidic system. The effects of five clinically relevant antibiotics (trimethoprim-sulfamethoxazole, piperacillin-tazobactam, meropenem, imipenem, and cefiderocol) were evaluated under dynamic conditions at sub-inhibitory concentrations (0.5 × Minimum Inhibitory Concentration (MIC)) and on preformed biofilm at inhibitory concentrations (10 × MIC). Non-CF isolates displayed faster early adhesion than CF isolates, whereas mature biofilm biomass was comparable between groups. If early adhesion did not predict mature biofilm biomass, dynamic biofilm coverage under flow conditions correlated with static mature biofilm levels. Sub-inhibitory antibiotic concentrations failed to prevent initial adhesion and elicited three distinct responses: biofilm formation enhancement (piperacillin-tazobactam, meropenem, imipenem), no effect (trimethoprim-sulfamethoxazole), or biofilm reduction (cefiderocol). Exposing mature biofilm to 10 × MIC identified trimethoprim-sulfamethoxazole and cefiderocol as the most effective agents in biofilm biomass reduction, whereas carbapenems and piperacillin-tazobactam were less effective. These findings provide new insights into *A. xylosoxidans* biofilm biology and may help guide therapeutic strategies for infections caused by this emerging, increasingly drug-resistant pathogen.

## Highlights

• Faster adhesion for non-CF *versus* CF strains but origin-independent mature biofilm.
• Early adhesion does not predict mature biofilm biomass in *A. xylosoxidans*.
• Biofilm coverage under flow strongly correlates with static mature biofilm.
• Sub-MIC antibiotics can increase, reduce, or have zero effect on biofilm.
• Trimethoprim-sulfamethoxazole and cefiderocol effectively reduce mature biofilm.

## 1 Introduction

Cystic fibrosis (CF) was one of the first infectious diseases in which biofilm formation was recognized as a central component of its pathogenesis [1] and remains one of the most extensively studied biofilm-related infections [2]. Since then, biofilms have become increasingly involved in a wide variety of non-CF infections, including infective endocarditis, chronic wounds, urinary tract infections, pharyngitis, laryngitis, and otitis media [2,3]. This broad clinical relevance raises the question of whether biofilm-related traits differ according to the context of infection in which pathogens evolve, particularly regarding CF airways, characterized by intense selective pressures, compared with other clinical settings. Among the opportunistic pathogens capable of causing persistent infections in both CF and non-CF patients, *Achromobacter* spp. - obligately aerobic, non-fermenting Gram-negative bacilli of the order *Burkholderiales* and family *Alcaligenaceae* [4] - have emerged as clinically relevant opportunistic pathogens due to their intrinsic antibiotic resistance and ability to form biofilm [5]. Within the 22 recognized species of the genus, *Achromobacter xylosoxidans* is by far the most commonly isolated species in both CF and non-CF patients [[6], [7], [8], [9], [10]].

Although biofilm formation has been proposed as a key trait in *A. xylosoxidans* [11], data directly comparing the biofilm-forming capacity of isolates from CF *versus* non-CF origins remain limited. To date, only one study has addressed this question and reported no significant difference between the two clinical groups [11]. However, these conclusions relied on crystal violet (CV) staining, a high-throughput assay commonly used to quantify total biofilm biomass that presents well-recognized methodological limitations, particularly in the early stages of biofilm development [12]. Static CV assays are subject to biases related to nutrient depletion and the accumulation of metabolic waste products, which may influence adhesion, early biofilm architecture, and quantification [13]. Furthermore, the inclusion of multiple *Achromobacter* species may have confused interpretation by introducing interspecies variability, independent of clinical origin.

To overcome these limitations, we compared biofilm formation in a well-defined collection of *A. xylosoxidans* isolates, of CF and non-CF origin, using complementary static and dynamic methodologies tailored to capture the distinct sequential stages of biofilm development.

Another hallmark of the *Achromobacter* spp. is their high level of intrinsic antimicrobial resistance, which severely limits therapeutic options [14]. First-line active agents typically include trimethoprim-sulfamethoxazole (TRS), piperacillin-tazobactam (PIT), meropenem (MER), and imipenem (IMI) [[14], [15], [16], [17], [18]], but their effectiveness is often compromised by acquired resistance mechanisms such as β-lactamase production, efflux pump overexpression, and resistance-associated mutations [19]. The emergence of multidrug-resistant (MDR) isolates has prompted increasing use of second-line agents such as cefiderocol (FDC), which shows potent activity against *A. xylosoxidans* [20]. Biofilm-associated tolerance further reduces antibiotic efficacy, even when planktonic susceptibility is preserved, contributing to the persistence of *Achromobacter* infections in both CF [21] and non-CF patients [22].

In this context, the present study aimed to characterize and compare early and mature biofilm formation in a collection of 57 *A. xylosoxidans* isolates from CF and non-CF origins. We additionally examined the effects of five clinically relevant antibiotics - four first-line agents (TRS, PIT, MER, IMI) and one second-line agent (FDC) - on biofilm behavior in microfluidic conditions.

## 2 Materials and methods

### 2.1 Bacterial strains

A total of 57 clinically documented *A. xylosoxidans* isolates (one per patient) were included: 28 from sputum samples from CF patients and 29 from non-CF patients. The non-CF isolates originated from various specimen types: 14 respiratory samples, five ear, nose and throat (ENT) samples, three tissue or wound biopsies, three blood cultures, one rectal screening, one ocular sample, one catheter, and one bone biopsy. Isolates were collected between 2012 and 2021 during routine diagnostic workflow at *i)* the CF centers at Paris and Montpellier University Hospitals and *ii)* Montpellier and Nîmes University Hospitals and the General Hospital of Alès-Cévennes for non-CF patients. The 57 isolates in the study had been identified as *A. xylosoxidans* by *nrdA* gene sequencing and phylogenetic analysis as previously described [9]. Their genetic diversity (15 distinct *nrdA* alleles) is presented in Supplementary Table 1. All strains were stored at - 80 °C in Trypticase-Soy broth supplemented with glycerol.

### 2.2 Study design

**Fig. 1:**
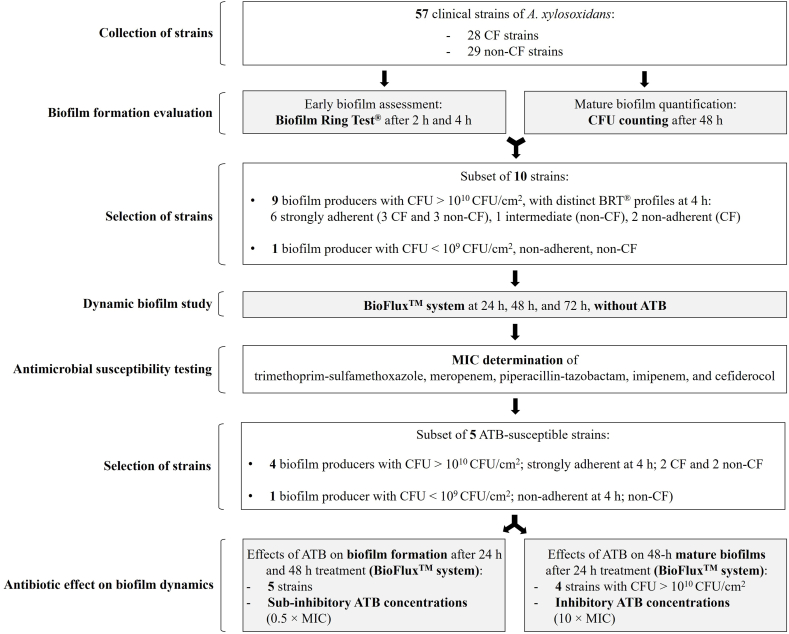
Flowchart showing the main stages of the study. Methods used to study *A. xylosoxidans* biofilm are shown in light gray boxes. Abbreviations: ATB, antibiotic; BRT®, Biofilm Ring Test®; CF, cystic fibrosis; CFU, colony-forming unit; MIC, minimum inhibitory concentration.

The study workflow is summarized in Fig. 1.

All 57 strains were assessed for initial bacterial adhesion/early biofilm formation by immobilizing microbeads [23] using the Biofilm Ring Test® (BRT®; BioFilm Control, Saint-Beauzire, France) and for mature biofilm formation using colony-forming unit (CFU) enumeration after 48 h of static incubation.

A subset of ten strains was analyzed in a continuous-flow microfluidic system (BioFlux™ 200, Fluxion Bioscience Inc., Alameda, CA, USA) [24], which, like other dynamic biofilm models, allows the study of biofilm development under flow and shear stress conditions in an attempt to more closely approximate *in vivo*-like dynamics compared to static methods. Strains were selected to represent the diversity of biofilm phenotypes observed under static conditions: nine biofilm producers with CFU counts > 10^10^ CFU/cm^2^ and displaying variable early adhesion profiles in the BRT® [six strongly adherent (three CF and three non-CF isolates), two non-adherent (CF), one intermediate (non-CF)], and one non-CF biofilm-producing strain (CFU counts < 10^9^ CFU/cm^2^, non-adherent at 4 h). Biofilm dynamics were monitored at 24, 48, and 72 h. Results at 48 h under flow were compared with those obtained for mature biofilms under static conditions. Antimicrobial susceptibility testing was performed for these ten strains.

From this panel, five strains (three non-CF and two CF) that were susceptible to all the antibiotics tested were used to evaluate the impact of sub-inhibitory concentrations (0.5 × Minimum Inhibitory Concentration (MIC)), on dynamic biofilm formation. To assess antibiotic activity against preformed biofilms, the four strong or moderate producers were exposed to high antibiotic concentrations (10 × MIC) for 24 h following 48 h of biofilm development in the BioFlux™ system.

### 2.3 Antimicrobial susceptibility testing

Antimicrobial susceptibility testing was performed on the ten selected *Achromobacter* strains. Inhibition zone diameters were determined by the disk diffusion method on Mueller–Hinton agar (Becton Dickinson, Le Pont-de-Claix, France) using TRS, PIT, MER, and IMI disks (Bio-Rad, Marnes-La-Coquette, France). Results were interpreted according to 2026 European committee on antimicrobial susceptibility testing (EUCAST) clinical breakpoints [25], except for IMI, for which tentative breakpoints from Clinical and Laboratory Standards Institute M45 (4^th^ edition) were applied (<18 mm: Resistant; 18-23 mm: Intermediate; ≥24 mm: Susceptible), due to the lack of specific EUCAST breakpoints [26]. MICs were subsequently determined for strains susceptible to all the antibiotics tested, using broth microdilution in cation-adjusted Mueller–Hinton broth (CA-MHB) (Becton Dickinson), according to EUCAST recommendations and ISO standard 20776-1 (https://www.eucast.org/clinical_breakpoints, accessed on 14^th^ March 2026). For PIT, MICs were determined using a fixed tazobactam concentration of 4 mg/L. TRS MICs were assessed using a ratio of 1:19 trimethoprim-to-sulfamethoxazole, with results expressed as trimethoprim concentrations. FDC MICs were determined by the French National Reference Centre for Antibiotic Resistance (Besançon, France) using iron-depleted CA-MHB, as previously described [20]. For antibiotics lacking EUCAST breakpoints (IMI, FDC), MICs were interpreted according to the 2025 Antibiogram Committee of the French Society of Microbiology (CA-SFM) non-species-related pharmacokinetic/pharmacodynamic breakpoints [18].

Quality controls were performed using *Pseudomonas aeruginosa* ATCC 27853 for all antibiotics, except for TRS, for which *Escherichia coli* ATCC 25922 was used (data not shown).

All MICs were determined in duplicate (Supplementary Table 1).

### 2.4 Kinetics of early biofilm formation

Early adhesion was assessed using the BRT® (BioFilm Control), according to the manufacturer's instructions [23]. This method measures the mobility of superparamagnetic microbeads under a magnetic field, reflecting the degree of bacterial immobilization during the early stages of biofilm formation.

Bacterial suspensions adjusted to an optical density at 600 nm (OD_600_) of 0.1 ± 0.05 were inoculated into 96-well microtiter plates (Falcon, Corning, USA) containing magnetic beads and Brain Heart Infusion (BHI) medium, then incubated at 37 °C without shaking. At 2 h and 4 h, 100 μL of contrast liquid was added to each well, and plates were successively placed on a magnetic block and scanned using a dedicated optical reader (Epson Scanner modified for microplate analysis). Image analysis was performed using BFC Elements® 3.0 software (BioFilm Control), which generates a Biofilm Formation Index (BFI) ranging from 0 (complete bead immobilization; strong adhesion) to 20 (free beads; no adhesion). Negative controls (beads + BHI without bacterial suspension) were included in each experiment. Two independent experiments were performed, each in duplicate, for each strain and incubation time.

Strains were classified into three categories based on the mean BFI values at 4 h, as described by Boudet et al. [27]: non-adherent (mean BFI ≈ 20), moderately adherent (mean BFI between 4 and 20), and strongly adherent (mean BFI <4). The 4-h time point was selected to discriminate fast from delayed adhesion whilst minimizing early-stage variability (Supplementary Table 1).

### 2.5 Colony-forming unit counts

Standardized bacterial suspensions (OD_600_ = 0.1 ± 0.05) were inoculated into 48-well microtiter plates (Falcon) and incubated at 37 °C for 48 h. After incubation, the supernatants were carefully removed, and the wells were gently washed with sterile phosphate-buffered saline (PBS) to eliminate planktonic cells. The remaining biofilm-associated cells were detached by sonication (2 × 15 min at 40 kHz) separated by a vortexing stage and resuspended in a final volume of 1 mL. Sonication conditions were optimized in preliminary experiments to ensure efficient biofilm detachment without decreasing viability. Ten-fold serial dilutions of the recovered suspensions were prepared in sterile PBS, and 10 μL of the appropriate dilutions were plated on Luria-Bertani agar using the drop plate method. Plates were incubated at 37 °C for 24 h or until countable colonies were formed. Results were first expressed as CFU/mL and subsequently normalized to the surface area of the well (∼0.95 cm^2^) to obtain CFU per surface unit (CFU/cm^2^) more appropriate for surface-associated biofilms. A negative control consisting of non-inoculated wells processed identically was systematically included to control for contamination. All assays were performed in duplicate (Supplementary Table 1).

### 2.6 Biofilm formation in a microfluidic dynamic system

Biofilm development under dynamic conditions, with or without antibiotics, was assessed using the BioFlux™ 200 microfluidic system (Fluxion Bioscience Inc.) with BHI medium (Sigma-Aldrich, Saint-Quentin-Fallavier, France), as previously described [13,28]. The device consists of a 48-well plate containing 24 inflow-outflow well pairs interconnected by microchannels, allowing real-time microscopic monitoring under controlled flow [24].

Overnight cultures grown in BHI at 37 °C with shaking (220 rpm) were adjusted to an OD_600_ of 0.1 ± 0.05 after two successive 1:100 dilutions in fresh BHI medium.

Before inoculation, channels were primed with 500 μL of BHI under 1 dyne/cm^2^ for 10 min. Bacterial suspensions were then introduced into inflow wells and incubated at 37 °C under a continuous flow of 0.2 dyne/cm^2^, a shear stress previously shown to support stable biofilm formation in microfluidic models [13].

Biofilm growth was evaluated: *i)* without antibiotics: after 24, 48, and 72 h of incubation, and *ii)* with antibiotics, according to two conditions, at antibiotic concentrations based on previous studies [13]:

• Sub-inhibitory conditions - antibiotic concentration corresponding to 0.5 × MIC in BHI broth, to assess the *in vitro* effect on biofilm formation after 24 h and 48 h;
• Inhibitory conditions - antibiotic concentration corresponding to 10 × MIC applied after 48 h of biofilm growth, followed by 24 h of exposure to assess activity against established biofilms.

All experiments were performed in triplicate (Supplementary Table 1).

After incubation, biofilm architecture was visualized using a Leica DM IRB inverted fluorescence microscope (Leica Biosystems, Nanterre, France) equipped with a CoolSNAP FX black-and-white camera (Roper Scientific, Trenton, NJ, USA). Images were acquired with MetaVue™ software (Molecular Devices, Sunnyvale, CA, USA).

Quantitative biofilm coverage was measured with ImageJ® software (National Institutes of Health, Bethesda, MD, USA). Images were processed in 16-bit grayscale format, and thresholds were adjusted to accurately delineate bacterial structures. The percentage of channel surface covered by biofilm was calculated using the “Analyze Particles” function, as described elsewhere [13,28].

### 2.7 Statistical analysis

Analyses were performed using GraphPad Prism version 8.0 (GraphPad Software, San Diego, CA, USA) and R version 4.4.2 (R Foundation for Statistical Computing, Vienna, Austria). Model assumptions were assessed by examining residual distribution and variance homogeneity. Normality was evaluated using the Shapiro-Wilk test and Q-Q plots, and homoscedasticity using Levene's test.

The non-parametric Mann-Whitney test was used to compare CF and non-CF strains for *i)* 4-h adhesion (median BFI values), *ii)* viable mature biofilm biomass under static conditions (median CFU/cm^2^), and *iii)* dynamic mature biofilm formation without antibiotics (biofilm coverage overall and at 24, 48, and 72 h). Within non-CF strains, differences according to sample type (respiratory, ENT, skin and soft tissue biopsy, blood culture) were assessed using Kruskal-Wallis tests for *i)* 4-h adhesion (BFI values) and *ii)* viable mature biofilm production under static conditions (CFU counts). Correlations between biofilm-related phenotypes were assessed using Spearman's rank correlation, including *i)* the relationship between 4-h adhesion and mature static biofilm at 48 h (overall, among CF strains, and among non-CF strains) and *ii)* the relationship between static *versus* dynamic mature biofilm formation at 48 h for a subset of strains.

The effects of sub-inhibitory antibiotic concentrations on *A. xylosoxidans* biofilm formation under dynamic conditions were analyzed using a two-way analysis of variance (ANOVA) to evaluate the effects of antibiotic treatment, bacterial strain, and their interaction on biofilm production (%). Two-way ANOVA was applied without Box-Cox power transformation when model assumptions were satisfied (24 h conditions), or after a Box-Cox power transformation to improve residual normality and variance homogeneity (48 h conditions).

The effects of antibiotics on 48-h preformed biofilms under dynamic conditions were analyzed using a two-way non-parametric permutational ANOVA with the aovperm function in R (permuco package) [29], because model assumptions remained unmet despite the application of a Box-Cox power transformation.

Post-hoc comparisons were performed using estimated marginal means (emmeans package [30]). Linear contrasts compared each antibiotic to the no-antibiotic control, with *p*-values adjusted using Dunnett's procedure to control the family-wise error rate. Statistical significance was set at α = 0.05 and reported as follows: *p* < 0.05 (∗), *p* < 0.01 (∗∗), and *p* < 0.001 (∗∗∗).

## 3 Results

### 3.1 Differential early biofilm formation between CF and non-CF *A. xylosoxidans* strains

**Fig. 2:**
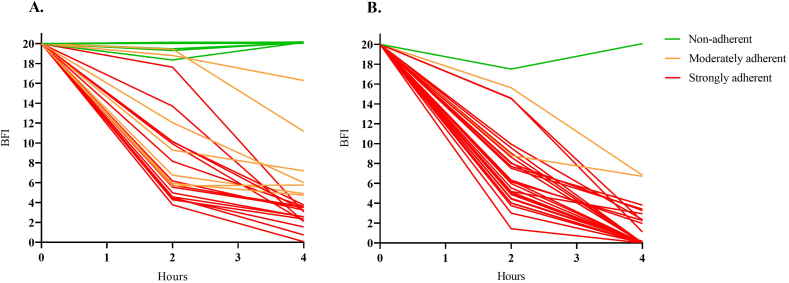
Dynamics of early biofilm formation for the 57 clinical strains of *A. xylosoxidans* of the study determined using the BioFilm Ring Test®. Biofilm formation index (BFI) values at 2 h and 4 h for *A. xylosoxidans* strains of CF origin (**A**) and non-CF origin (**B**). Strains were classified into three categories according to their mean BFI values at 4 h: non-adherent (mean BFI values between 20.13 and 20.07; green lines), moderately adherent (mean BFI values between 16.30 and 4.75; orange lines), and strongly adherent (mean BFI values between 3.81 and 0; red lines) [27]. Non-CF strains adhered significantly faster than CF strains (Mann-Whitney test, *p* < 0.0001). (For interpretation of the references to colour in this figure legend, the reader is referred to the Web version of this article.)

At 4 h, most of the 57 *A. xylosoxidans* strains (n = 41, 71.9%) were classified as strongly adherent according to the BRT®, whereas seven (12.3%) were non-adherent and nine (15.8%) were moderately adherent. Early adhesion greatly differed according to the clinical origin of strains. Almost all non-CF strains were strongly adherent (26/29, 89.7%) compared with CF strains (15/28, 53.6%). Only one non-CF strain (3%), recovered from a blood culture, failed to adhere after 4 h, compared with six CF strains (21.4%). Moderate adhesion was observed in two non-CF strains (7%) and seven CF strains (25%). Overall, non-CF strains adhered significantly faster than CF strains (median BFI: CF = 3.69 *vs.* non-CF = 0.07; Mann-Whitney test, *p* < 0.0001) (Fig. 2). Within the non-CF group, early adhesion did not differ significantly according to specimen type (respiratory, ENT, skin and soft tissue biopsy, blood culture samples; median BFI: 0, 0, 0, and 2.48, respectively; Kruskal-Wallis test, *p* = 0.3879, data not shown).

### 3.2 Similar mature biofilm formation in CF and non-CF *A. xylosoxidans* strains under static conditions

Mature biofilm formation after 48 h revealed substantial variability across strains, with viable counts ranging from 4.5 × 10^7^ to 6.3 × 10^11^ CFU/cm^2^ (mean: 3.3 × 10^10^; median: 5.9 × 10^9^). CF strains ranged from 4.5 × 10^7^ to 1.9 × 10^11^ CFU/cm^2^ (mean: 2.6 × 10^10^; median: 1.0 × 10^10^), whereas non-CF strains ranged from 1.6 × 10^8^ to 6.3 × 10^11^ CFU/cm^2^ and showed a slightly higher mean (4.1 × 10^10^ CFU/cm^2^) but a lower median (4.2 × 10^9^ CFU/cm^2^) (Supplementary Table 1).

**Fig. 3:**
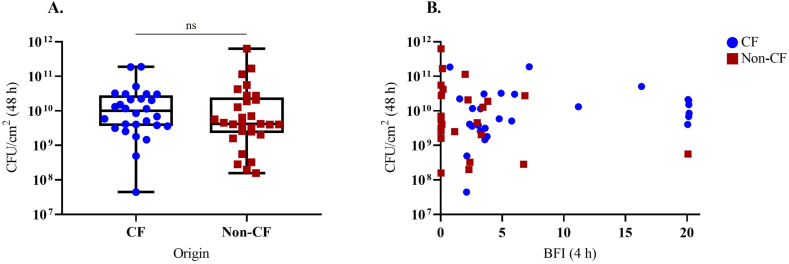
Comparison of 48 h mature biofilm biomass by clinical origin and its relationship with early adhesion. (**A**) Box plot showing similar mature biofilm formation in CF and non-CF *A. xylosoxidans* strains (Mann-Whitney test, *p* = 0.3191). (**B**) No significant correlation was found between early biofilm formation (BFI values) and mature biofilm biomass (CFU counts) (Spearman's test, *p* = 0.4511; r = 0.1018). Abbreviations: BFI, biofilm formation index; CF, cystic fibrosis; CFU, colony-forming unit; ns, not significant.

All *A. xylosoxidans* strains, including those classified as non-adherent at 4 h with BRT®, were able to form mature biofilms after 48 h. In contrast to early adhesion, mature biofilm biomass did not differ significantly between CF and non-CF strains (Mann-Whitney test, *p* = 0.3191) (Fig. 3A). No significant differences were detected among non-CF strains according to specimen type (respiratory, ENT, skin and soft tissue biopsy, blood culture; median CFU/cm^2^: 5.4 × 10^9^, 2.0 × 10^9^, 4.0 × 10^9^, 5.6 × 10^8^, respectively; Kruskal-Wallis test, *p* = 0.4146, data not shown).

### 3.3 Early adhesion does not predict mature biofilm formation in *A. xylosoxidans*

Early adhesion and mature biofilm biomass showed no consistent relationship. While the only non-CF strain lacking early adhesion revealed lower counts of sessile bacteria in mature biofilm (<10^9^ CFU/cm^2^), the six CF strains that were non-adherent at 4 h in the BRT® assay formed mature biofilms >10^9^ CFU/cm^2^ after 48 h. Among the CF strains, two strains that adhered quickly at 4 h produced mature biofilms <10^9^ CFU/cm^2^. Among the non-CF strains, the five strains associated with lower counts of sessile bacteria in mature biofilm (<10^9^ CFU/cm^2^) included three strong early biofilm producers, one moderate, and one non-adherent strain (Supplementary Table 1; Fig. 3B).

Spearman analysis confirmed the absence of correlation between early adhesion (4 h-BFI values) and mature biofilm biomass (CFU counts) (overall: *p* = 0.4511; r = 0.1018), for either CF (*p* = 0.3154; r = 0.1968) or non-CF strains (*p* = 0.7035; r = −0.074) (Fig. 3B). These results show that early adhesion ability of *A. xylosoxidans* strains does not predict their capacity to form mature biofilm. Various biofilm dynamics have been described here: some initially weakly adherent strains developed substantial mature biofilms, whereas others that adhered quickly did not necessarily produce high mature biofilms biomass.

### 3.4 Comparable biofilm formation under flow conditions in CF and non-CF *A. xylosoxidans* strains

To further characterize biofilm formation in *A. xylosoxidans*, ten strains displaying diverse phenotypes under static conditions were analyzed in the BioFlux™ 200 system. Nine isolates exhibited substantial mature biofilms at 48 h (>10^10^ CFU/cm^2^), including five CF isolates (three with strong early adhesion at 4 h [Ax P3-52, II 1, V 81] and two non-adherent at 4 h [III 81, Ax P17-73]) and four non-CF isolates (three strongly adherent [1195, 3413, 5132] and one moderately adherent at 4 h [1009466247]). The tenth isolate (1012239065, non-CF) was distinguished by the formation of a lower mature biofilm biomass (<10^9^ CFU/cm^2^) and the absence of early adhesion at 4 h (Supplementary Table 1).

**Fig. 4:**
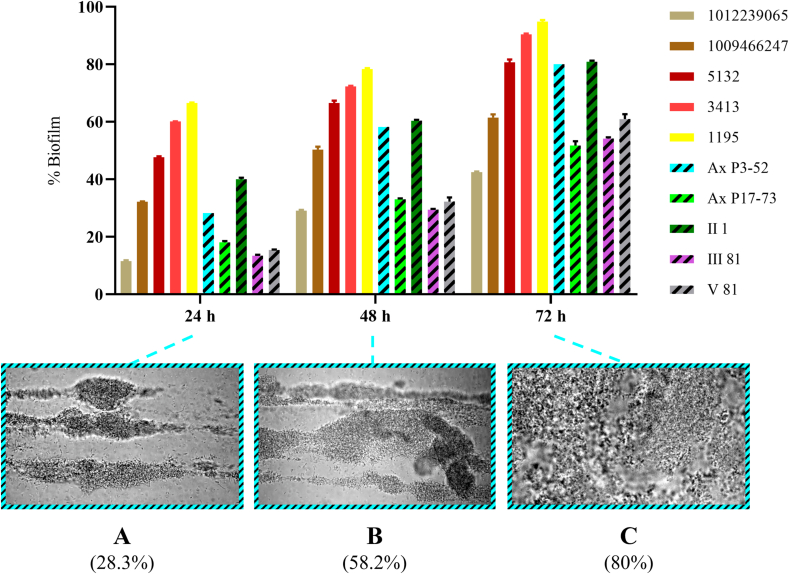
Biofilm formation kinetics of CF and non-CF *A. xylosoxidans* strains in the BioFlux™ dynamic system. Biofilm formation was expressed as the percentage of surface coverage after 24, 48, and 72 h of incubation in BHI medium. Data are presented as means ± standard deviations from three independent experiments. Hatched bars represent strains of CF origin. Panels **A-C** show representative images of biofilm development by the CF strain Ax P3-52 (strong early adhesion and high mature biomass at 48 h) at 24, 48, and 72 h, respectively, with the corresponding percentages of surface coverage indicated for each time point.

Under flow conditions, biofilm formation increased steadily over time ranging from 11.5 to 66.6% (mean 33.3%) at 24 h, 29-78.3% (mean 51%) at 48 h, and 42.5-94.9% (mean 69.8%) at 72 h. Kinetics were strain-specific and remained consistent across time points: low producers at 24 h (e.g., 1012239065) remained low at later time points, whereas high producers (e.g., 1195) maintained higher biofilm coverage throughout the assay (Fig. 4). To investigate the impact of clinical origin on biofilm formation under dynamic conditions, biofilm coverage was compared between three CF (Ax P3-52, II 1, and V 81) and three non-CF strains (1195, 3413, and 5132) exhibiting similar behavior under static conditions (strong early adhesion and moderate or high viable mature biomass at 48 h). No significant differences in biofilm coverage were observed between CF and non-CF strains at any time point (Mann-Whitney test: 24 h, *p* = 0.1; 48 h, *p* = 0.1; 72 h, *p* = 0.2). A significant correlation was observed between 48-h CFU counts under static conditions and biofilm coverage at 48 h under flow (Spearman's test, *p* = 0.0032), indicating coherence between the two methodologies.

### 3.5 Sub-inhibitory antibiotic concentrations affect biofilm formation in *A. xylosoxidans* differentially

**Fig. 5:**
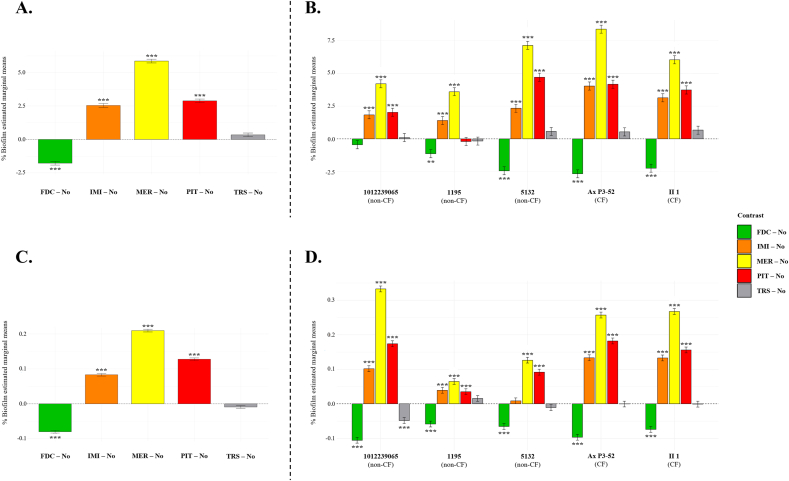
Effect of sub-inhibitory concentrations of antibiotics on biofilm formation in *A. xylosoxidans* under dynamic conditions. Histograms represent the estimated marginal means of each “ATB – No” contrast, corresponding to the difference in surface coverage percentage between sub-inhibitory antibiotic conditions (0.5 × MIC) (“ATB”) and antibiotic-free conditions (“No”) after 24 h (**A** and **B**) and 48 h (**C** and **D**) of incubation. Panels **A** and **C** illustrate the overall antibiotic effect across all strains combined, whereas panels **B** and **D** show strain-specific responses. In the post-hoc statistical model, the “ATB – No” contrast was tested against 0: positive values indicate increased biofilm production relative to the control condition, whereas negative values indicate decreased biofilm production. Error bars represent the standard errors of the estimated marginal means. Statistical significance was defined as follows: *p* < 0.05 (∗), *p* < 0.01 (∗∗), and *p* < 0.001 (∗∗∗). Abbreviations: ATB, antibiotic; FDC, cefiderocol; IMI, imipenem; MER, meropenem; ns, not significant; PIT, piperacillin-tazobactam; TRS, trimethoprim-sulfamethoxazole.

The MIC values of the antibiotics being tested against the *A. xylosoxidans* under study are presented in Supplementary Table 1, with identical results obtained across duplicate experiments. Five antibiotic-susceptible strains were selected for subsequent analyses based on their contrasting biofilm phenotypes, ranging from strong early adhesion with moderate or high mature biomass (two non-CF and two CF strains) to non-adherent and weak biofilm production (one non-CF strain). All strains formed biofilms in the presence of sub-inhibitory concentrations (0.5 × MIC) at 24 h and 48 h, showing a progressive increase in biofilm biomass overtime: none of the five antibiotics fully prevented initial adhesion (Supplementary Table 1). However, three distinct profiles emerged: *i)* Biofilm stimulation: PIT, MER, and IMI significantly increased biofilm formation at both 24 h (Fig. 5A and B) and 48 h (Fig. 5C and D) for nearly all strains, except for PIT on non-CF strain 1195 at 24 h (Fig. 5B), and IMI on non-CF strain 5132 at 48 h (Fig. 5D) for which no significant change was observed; *ii)* No effect: TRS had no significant impact at either time point, except for the non-CF strain 1012239065, where it reduced biofilm formation at 48 h (Fig. 5D); *iii)* Biofilm inhibition: FDC significantly reduced biofilm formation at 24 h and 48 h in all strains, except for the non-CF strain 1012239065 at 24 h for which no significant effect was observed (Fig. 5B).

### 3.6 Mature *A. xylosoxidans* biofilm is most effectively reduced by trimethoprim-sulfamethoxazole and cefiderocol

**Fig. 6:**
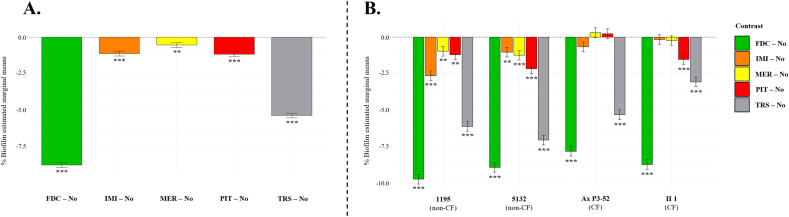
Antibiotic activity against preformed *A. xylosoxidans* biofilm under dynamic conditions. Histograms represent the estimated marginal means of each “ATB – No” contrast, corresponding to the difference in surface coverage percentage after 24 h of incubation between antibiotic conditions (10 × MIC) (“ATB”) and antibiotic-free conditions (“No”), on 48-h preformed *A. xylosoxidans* biofilms. Panel **A** illustrates the overall antibiotic effect across all strains combined, whereas panel **B** shows strain-specific responses. In the post-hoc statistical model, the “ATB – No” contrast was tested against 0: positive values indicate increased biofilm production relative to the control condition, whereas negative values indicate decreased biofilm production. Error bars represent the standard errors of the estimated marginal means. Statistical significance was defined as follows: *p* < 0.05 (∗), *p* < 0.01 (∗∗), and *p* < 0.001 (∗∗∗). Abbreviations: ATB, antibiotic; FDC, cefiderocol; IMI, imipenem; MER, meropenem; ns, not significant; PIT, piperacillin-tazobactam; TRS, trimethoprim-sulfamethoxazole.

The activity of antibiotics against preformed 48-h biofilm was evaluated in the four strong or moderate biofilm-forming strains (two CF, two non-CF) (Fig. 6). TRS and FDC consistently reduced mature biofilm biomass across all strains (*p* < 0.001). Among the other antibiotics, PIT reduced biofilm biomass in the two non-CF strains and in CF strain II-1, whereas MER and IMI produced only modest biofilm reduction and exclusively in the two non-CF strains. The two non-CF strains, notably, were the only ones to show significant biofilm reduction in response to all five antibiotics tested (Fig. 6; Supplementary Table 1).

## 4 Discussion

To the best of our knowledge, this study is the first to compare early and mature biofilm formation between CF and non-CF *A. xylosoxidans* isolates, and the first to investigate antibiotic-biofilm interactions using a microfluidic platform designed to better approximate *in vivo* conditions. Using complementary approaches, we showed that CF and non-CF isolates shared a strong ability to form biofilms despite distinct formation kinetics. We also identified TRS and FDC as the most promising antibiofilm agents, as both significantly reduced mature biofilms, while FDC additionally impaired biofilm formation.

### 4.1 *Achromobacter* spp. in biofilm-related diseases

Biofilm formation appears to be a widespread trait among *Achromobacter* isolates across diverse clinical settings. Most of the evidence available comes from CF isolates, which commonly form biofilms *in vitro* and contribute to long-term persistence in CF airways [6,11,19,[31], [32], [33], [34], [35], [36], [37], [38]]. However, non-CF isolates described in the literature - predominantly from respiratory samples [11,31,[39], [40], [41]], bloodstream infections [11,31], and ENT specimens [11,[42], [43], [44]] - also demonstrate a biofilm-forming capacity. The clinical relevance of *Achromobacter* biofilms in non-CF contexts is supported by *in vivo* evidence, including a chronic scalp infection strongly suggestive of biofilm involvement [45]. In addition, *Achromobacter* readily colonizes a wide range of medical devices, such as central venous catheters [46], contact lenses [[47], [48], [49]], urinary catheters [50], and prosthetic heart valves [22], thereby contributing to bloodstream infections, keratitis and corneal ulcers, catheter-associated urinary tract infections and infective endocarditis, respectively [22,[46], [47], [48], [49], [50]].

Together, these data highlight biofilm formation as a clinically relevant trait of *Achromobacter* sp. across CF and non-CF settings.

### 4.2 Distinct early kinetics but similar overall biofilm-forming ability in CF and non-CF strains

Given the clinical importance of *Achromobacter* biofilm on patient outcomes, we compared the biofilm-forming capacities of 28 CF and 29 non-CF strains, starting with early adhesion evaluated using the BRT®, previously used for *Staphylococcus aureus* [27] and *P. aeruginosa* [51], but applied for the first time here to strains of the *Achromobacter* genus. We observed significantly faster adhesion kinetics in non-CF strains, most of which formed early biofilms within 4 h, whereas several CF strains displayed delayed adhesion. Similar findings have been reported in another bacterial species using CV-based assays, with Biočanin et al. describing seven strong biofilm-producing *Stenotrophomonas maltophilia* strains (three non-CF and four CF), suggesting that these observations maybe explained, at least partly, by the higher motility of non-CF isolates [52]. However, these findings contrast with those of Filipic et al., who reported enhanced binding of 32 CF-derived *Achromobacter* strains to mucin, collagen, and, to a lesser degree, fibronectin relative to 37 non-CF strains [11]. This was interpreted as an adaptation to the CF lung environment, where mucus hypersecretion and extracellular matrix deposition (with collagen and fibronectin) are characteristic features [53]. Importantly, the CF strains in their study originated from respiratory samples, mainly from chronically colonized patients (24/32 strains), whereas non-CF strains were drawn from heterogeneous origins (respiratory samples, blood, nasal swabs, and ear swabs) and often transient infections. In our study, the CF group included a more balanced distribution of early and late isolates, with 17/28 potentially host-adapted late isolates and 11/28 early isolates recovered during primary colonization or transient infections. The non-CF group was also heterogeneous, including respiratory isolates from patients with chronic pulmonary disease as well as device-associated isolates, both of which may influence biofilm formation. In addition, direct comparison with our study is limited by methodological differences: Filipic et al. hypothesized that CF strains may be better adapted to the pulmonary environment, which probably contributes to their enhanced adhesion to lung-specific components [11]. They measured binding to host substrates using chemical CV-based assays that emphasize matrix binding and interactions with lung-specific components, thereby providing insights into the adaptation of CF strains to the respiratory environment. In contrast, our study used the BRT® which objectively quantifies bead immobilization on abiotic surfaces, providing a substrate-independent measure of early adhesion. In addition, we consistently used BHI medium recommended by the BRT® manufacturer whereas Filipic et al. used Luria-Bertani broth [54]. Given that culture media strongly influence gene expression and biofilm phenotypes [34,55], these differences may contribute to divergent findings and provide complementary perspectives. Furthermore, non-surface-attached bacterial aggregates are now widely recognized as biofilm structures and integral components of the biofilm life cycle [56]. These multicellular clusters, which form in mucus, sputum, and other host environments, share key features with surface-attached biofilms and can contribute to early adhesion processes. This aspect may partly explain discrepancies observed with the findings of Filipic et al. Indeed, the BRT® assay provides a global assessment of biofilm formation, capturing both surface-attached and unattached bacterial communities, whereas other adhesion assays - including those using mucin-, collagen-, or fibronectin-coated surfaces [11] - typically include washing steps that remove free floating aggregates. Despite differences during early adhesion, mature biofilm production under static conditions did not differ between CF and non-CF strains, consistent with Filipic et al. [11]. Likewise, under flow conditions, biofilm coverage showed no significant difference and correlated strongly with static 48-h mature biomass, indicating coherence across methodologies. These results support the view that biofilm formation in *Achromobacter* is primarily strain-dependent rather than determined by clinical origin, as previously suggested [57]. Chronic adaptation within CF airways may promote advantageous traits *in vivo* such as interaction with host matrix components without necessarily enhancing adhesion to abiotic surfaces.

Furthermore, a single culture medium (BHI) was used throughout the study to ensure comparability across strains, regardless of their CF or non-CF origin, while possibly not optimal for CF strains as alternative media, such as artificial sputum medium, which contains mucin, amino acids, and extracellular DNA are known to better approximate the CF lung environment [27]. However, as we conducted a comparative study, we evaluated strain behavior in fixed conditions, *i.e*., BHI medium for all strains, to avoid biasing comparisons by favoring conditions specifically adapted to CF strains which could have in turn disadvantaged non-CF strains.

The robustness of our findings is strengthened by the exclusive inclusion of *A. xylosoxidans* strains confirmed by *nrdA* sequencing combined with phylogeny [9], and by the relatively large number of strains (n = 57). Only four previous studies have included similar or larger isolate collections: [11], n = 60 *A. xylosoxidans* strains; [58], n = 68 *Achromobacter* spp. isolates; [59], n = 94 *A. xylosoxidans* isolates; and [19], n = 95 *Achromobacter* spp. isolates. Altogether, these results reinforce the concept that biofilm formation is a central, widespread feature of *A. xylosoxidans*, contributing to its persistence and epidemiological success across diverse clinical settings, by increasing tolerance to antimicrobial agents that would otherwise be effective against planktonic cells [6].

### 4.3 Variable antibiofilm activity of clinically relevant anti-*Achromobacter* antibiotics

Given the involvement of *A. xylosoxidans* in biofilm-associated infections, understanding the antibiofilm properties of the relevant antibiotics used to treat these infections is essential to optimize treatment strategies.

This study represents the second largest dataset in the literature in terms of both the number of *Achromobacter* strains and antibiotics tested, after Tom et al., among a total of 16 *in vitro* studies on antibiotic antibiofilm activities [58]. Unlike previous works, which did not simultaneously assess the inhibition of biofilm formation and activity against mature biofilm on the same strains, our study provides a more comprehensive *in vitro* evaluation of antibiofilm activity by examining both endpoints, focusing on five clinically relevant antibiotics: MER, IMI, PIT, TRS, and FDC. Although the antibiofilm activity of other antibiotics has been previously investigated towards strains of the *Achromobacter* genus - including agents to which *A. xylosoxidans* exhibits an expected resistant phenotype, such as colistin, aminoglycosides (tobramycin, amikacin, gentamicin), azithromycin, tetracycline, and β-lactams (ampicillin and aztreonam), as well as agents to which *A. xylosoxidans* is not intrinsically resistant, like chloramphenicol, fluoroquinolones (ciprofloxacin, levofloxacin), and β-lactams (amoxicillin–clavulanic acid, piperacillin, ceftazidime ± avibactam) - so far no studies have ever evaluated the antibiofilm activity of these five antibiotics under dynamic flow conditions in the *Achromobacter* genus.

MER was the only carbapenem evaluated, across three studies involving a total of eight strains. One study reported both inhibition of bacterial adhesion and biofilm formation [60]. Another study showed a reduction in biofilm formation without affecting bacterial adhesion. However, when MER was combined with CTV, the combination not only retained its antibiofilm activity but also exhibited significant anti-adhesion effects [61]. Additionally, one study demonstrated MER activity against mature biofilm [21]. In contrast, our results demonstrated that sub-inhibitory concentrations of MER consistently increased biofilm formation in all strains at 24 h and 48 h, whereas activity against mature biofilms was only observed in two non-CF strains, highlighting a strain-dependent effect.

PIT has been considered largely ineffective against mature *Achromobacter* biofilm based on high minimum biofilm eradication concentration (MBEC) in a study of three strains [6]. Here, PIT slightly reduced mature biofilm in three of four strains yet also increased biofilm formation at sub-inhibitory concentrations, again supporting a strain-dependent response.

TRS has been sparsely studied, with one report describing MBEC values far above the MICs of two strains, indicating limited activity against mature biofilm [21]. In our experiments, TRS showed no consistent effect at sub-inhibitory concentrations (except for a decrease observed in one non-CF strain), but significantly reduced mature biofilm in all strains, suggesting potential antibiofilm efficacy. This activity was further supported by a recent *in vivo* case report describing the antibiofilm effects of TRS in a chronic scalp infection [45].

Although the *in vitro* activity of FDC, a siderophore cephalosporin, against planktonic *Achromobacter* cells has recently been demonstrated [20], its antibiofilm activity in this genus remains unknown, as it is also the case for IMI.

Our results show that, similar to MER, IMI can increase biofilm formation at sub-inhibitory concentrations, whilst exhibiting only modest activity against mature biofilm in half the strains tested, all of non-CF origin. In contrast, FDC demonstrates potent *in vitro* antibiofilm activity, both reducing early biofilm formation at sub-inhibitory concentrations and preformed biofilm across all strains tested. To date, only one previous clinical case has suggested the *in vivo* antibiofilm activity of FDC in *Achromobacter* endocarditis [22].

Although MICs were determined in CA-MHB, or iron-depleted CA-MHB for FDC, antibiofilm assays were performed in BHI, a non-iron-depleted medium, which represents a potential limitation in interpreting FDC activity. However, since iron depletion does not accurately mimic *in vivo* conditions and previous studies have shown that iron availability has only a limited impact on FDC's efficacy against biofilm [62], this limitation is likely to have had only a minor influence on our findings. Among carbapenems, IMI appeared more favorable than MER, as MER was associated with greater biofilm formation in a larger number of strains at sub-inhibitory concentrations. Finally, the antibiofilm activity of PIT was generally comparable to that of the carbapenems. Although the number of strains tested under flow was limited, the combined data support TRS and FDC as the most promising antibiofilm agents against *A. xylosoxidans*, as neither promoted biofilm formation at sub-inhibitory concentrations and both significantly reduced established biofilms.

### 4.4 Species-dependent antibiofilm activity of antibiotics in other gram-negative pathogens

Based on the involvement of *A. xylosoxidans* in polymicrobial infections [[63], [64], [65]], we searched the literature for studies on antibiofilm activity of the studied antibiotics against other Gram-negative pathogens that may co-occur with *A. xylosoxidans*, particularly in CF infections. Regarding PIT, sub-MIC in *P. aeruginosa* PaO1 induced marked upregulation of biofilm-associated genes [66]. Although not investigated in our study, a similar mechanism may partly explain our observations in *A. xylosoxidans* exposed to sub-MIC levels of PIT, MER, and IMI. For TRS, promising antibiofilm activity was observed against *A. xylosoxidans* in our study, whereas the literature suggests strain-dependent antibiofilm activity [63,64] and concentration-dependent biofilm eradication in *S. maltophilia* [52,65]. In *Acinetobacter baumannii* (ATCC 17978), sub-inhibitory TRS concentrations were shown to repress CsuA/B pili expression and prevent biofilm formation promoting biofilm dispersal [67]. Whether a similar mechanism operates in our *A. xylosoxidans* non-CF strain 1012239065, which exhibited reduced biofilm formation under sub-inhibitory TRS concentrations, remains to be determined. Among carbapenems, MEM alone showed limited antibiofilm activity in *P. aeruginosa*, as reported here in *A. xylosoxidans*, but significantly improved biofilm eradication when combined with colistin (not tested on *A. xylosoxidans*) [68,69]. For IMI and comparative studies between IMI, FDC and PIT, species-dependent effects have been previously reported, with PIT consistently showing the lowest antibiofilm activity [62]. In six MDR species, FDC was superior to IMI in reducing biofilms [*P. aeruginosa* (93.6% *vs.* 49.3%), *S. maltophilia* (97.2% *vs.* 71.4%), and the *Burkholderia cepacia* complex (83% *vs.* 21.3%)], whereas similar activity was observed against *Klebsiella pneumoniae* (83.7% *vs.* 82.6%). On the other hand, FDC was less effective than IMI against *E. coli* (67.6% *vs.* 95.9%) and *A. baumannii* (80.9% *vs.* 92.9%). Increasing FDC concentrations further reduced biofilm biomass, although the effect plateaued at 16 mg/L [62]. Combined with the results of this study and our previous work on FDC susceptibility in *Achromobacter* spp., the wide antibiofilm activity of FDC observed across clinically relevant Gram-negative species is of particular interest in polymicrobial infections involving *A. xylosoxidans*. As stated for other species, FDC antibiofilm activity in *A. xylosoxidans* may be linked to two complementary mechanisms: *i)* the requirement of siderophores (largely produced by *A. xylosoxidans* [9]) for biofilm development [70] and *ii)* upregulation of siderophore transporters during biofilm formation [62], as both of these are affected during FDC therapy. Finally, direct observation by scanning electron microscopy of five sessile clinical *P. aeruginosa* strains showed that FDC and IMI treatments induced distinct effects, *i.e.* cell elongation and filamentation without altering matrix structure, suggesting inhibition of bacterial division for FDC, and increased matrix production with bacterial cells becoming shorter and rounder for IMI. Interestingly, the combination of FDC and IMI markedly reduced cell viability, producing synergistic bactericidal effects against all strains and synergistic biofilm eradication in one isolate [71]. These observations further highlight the potential interest of combination therapies, especially the combination of FDC with IMI, for dealing with biofilm-associated infections.

## 5 Conclusion

The strong impact of experimental design and scoring methods on biofilm-related conclusions underscores the need to apply methodologies specifically adapted to the biological question being addressed. In this context, the combined use of the BRT® to investigate early adhesion and dynamic flow-based system to monitor biofilm development over time, two approaches not previously applied to *Achromobacter*, provided more physiologically relevant and complementary insights into biofilm behavior in this genus. Using these approaches, we demonstrated that the clinical origin of *Achromobacter* strains does not predict their capacity to form biofilm, supporting a strain-dependent rather than an origin-dependent phenotype. This study also provides the first evidence of IMI and FDC's antibiofilm activity against *Achromobacter* and indicates that TRS and FDC have the most consistent antibiofilm activity, affecting both biofilm formation and mature biofilms, whereas carbapenems and PIT are less effective and more prone to enhancing biofilm formation at sub-inhibitory concentrations. However, the underlying mechanisms of antibiotic interferences with biofilm development, as the identification of affected stages, still have to be deciphered. Our investigations conducted on a limited number of *A. xylosoxidans* thus provide a basis for larger studies focusing on the most promising agents identified here, TRS and FDC, including a broader range of strains and *Achromobacter* species, as well as analyses of biofilm structural changes under antibiotic exposure. Finally, while FDC is not currently part of standard therapy for *Achromobacter* infections, it is considered in certain cases of therapeutic failure or limited treatment options [20], representing clinical situations where antibiofilm activity could constitute an additional advantage.

Altogether, our findings emphasize the need to develop robust *in vitro* tools capable of evaluating antimicrobial activity under conditions that better mimic the *in vivo* environment, which could ultimately help physicians make better-informed therapeutic decisions when dealing with infections caused by *Achromobacter* spp.

## Declaration of generative AI and AI-assisted technologies in the manuscript preparation process

During the preparation of this work, the authors used DeepL and ChatGPT to improve the language and readability. After using these tools, the authors reviewed and edited the content as required and take full responsibility for the content of this article.

## Funding

This research did not receive any specific grant from funding agencies in the public, commercial, or not-for-profit sectors.

## CRediT authorship contribution statement

**Vincent Jean-Pierre:** Conceptualization, Formal analysis, Methodology, Software, Writing – original draft. **Pauline Sorlin:** Conceptualization, Methodology, Writing – review & editing. **Catherine Dunyach-Remy:** Conceptualization, Methodology, Writing – review & editing. **Florian Salipante:** Formal analysis, Writing – review & editing. **Raphaël Chiron:** Resources, Writing – review & editing. **Jean-Philippe Lavigne:** Supervision, Validation, Writing – review & editing. **Cassandra Pouget:** Conceptualization, Formal analysis, Investigation, Methodology, Software, Writing – review & editing. **Hélène Marchandin:** Conceptualization, Methodology, Project administration, Validation, Visualization, Writing – review & editing.

## Declaration of competing interest

The authors declare that they have no known competing financial interests or personal relationships that could have appeared to influence the work reported in this paper.

## Data Availability

All data generated or analyzed during this study are included in this article (and its Supplementary Table 1).
